# Can leisure and entertainment lifestyle promote health among older people living alone in China?—A simultaneous equation approach

**DOI:** 10.3389/fpubh.2022.967170

**Published:** 2022-09-29

**Authors:** Yinghua Qin, Jingjing Liu, Rizhen Wang, Xinye Qi, Shengchao Jiang, Jiacheng Li, Pengfei Guo, Qunhong Wu

**Affiliations:** ^1^Department of Social Medicine, School of Public Health, Health Management College, Harbin Medical University, Harbin, China; ^2^Department of Health Economy and Social Security, College of Humanities and Management, Guilin Medical University, Guilin, China

**Keywords:** leisure and entertainment life, older people living alone, multidimensional health, simultaneous equations approach, China

## Abstract

**Objectives:**

With the surging number of older people living alone, their lifestyles and health status have aroused increasing concern. This study aims to investigate whether a leisure and entertainment lifestyle (LEL) can improve the multidimensional health among older people living alone and try to identify the latent mechanisms among them.

**Method:**

For this purpose, we extracted data from the Chinese General Social Survey (CGSS) and established a simultaneous equations model, comprising ordinary least square regression (OLS), two-stage least squares (2SLS), and the mediating effect model.

**Results:**

Older people living alone in China reported relatively better mental health status (3.64 ± 1.07), followed by physical health (3.41 ± 1.26) and social health (2.75 ± 1.18). In the OLS model, LEL significantly improved the social health of older people living alone (β = 0.054, *P* < 0.01), followed by physical health (β = 0.042, *P* < 0.01) and mental health (β = 0.027, *P* < 0.01). After endogenous tests from 2SLS model and robustness tests, we found that more active LEL was associated with higher levels of physical health and mental health. However, LEL had no significant effect on the improvement of the social health of the older people living alone. Using the mediation analysis, exercise efficacy partially mediated the relationship of LEL with physical health and mental health, and the ratios were 19.75 and 24.85%, respectively. Similarly, positive life attitudes partially mediated the relationship between LEL and physical health, and LEL and mental health, with ratios of 10.65 and 26.83%, respectively.

**Conclusion:**

Our findings suggested that LEL is significantly associated with better physical and mental health for older people living alone in China, and the association is mediated by exercise efficacy and positive attitudes toward life. Promoting more leisure and recreational activities, upgrading exercise efficacy, and encouraging positive life attitudes are necessary health promotion measures in active aging policies for the wellbeing of older people living alone.

## Background

The world is witnessing a rapid demographic shift toward population aging. Therefore, investigating and understanding the health and well-being of older people is of priority, especially in China. As home to one-fifth of the global population, China is a typical example of a country with an aging trend, with older people accounting for 14.2% of the total population in 2021 (1). With the implementation of the family planning policy in China, the traditional nuclear family structure has changed, and the family sizes have reduced, thus breaking the traditional Chinese family model of three or even four generations living together. Economic development and urbanization have pushed young people to work away from home (2–4). Moreover, the older people and children in modern society demand their own “free space” influenced by the Western culture. Because of these reasons, there is an increasing number of empty-nest older people living alone in China (5–7). However, older people living alone are different from empty-nest older people. The latter generally refers to older couples living together and taking care of and helping each other. In contrast, for older people living alone, not only have their children left home but they have also experienced widowhood or divorce or have always been single, this makes them more vulnerable than empty-nest older people (8). In China, where filial piety prevails, due to the absence or limitation in the quantity of family interactions, the older people living alone are at greater risk of long-term exclusion from social relations and are more likely to experience limited access to health services, which may pose a potential risk to their physical and mental health (9–12).

It is well-established that age-related conditions and, in particular, physical health, functional health, mental health, and psychosocial vulnerability are burdens for older people (13–16). This is related to multiple factors, including objective and subjective indicators, respectively (17). Although objective functional health is the main predictor of perceived health, empirical results on the associations between functional health and subjective health are often inconsistent, ranging from low and non-significant associations to highly significant associations (16, 18–22). Part of this heterogeneity may be based on the assessment of the expanded function in functional health, mainly leisure activities (23). The later stage of the life course is broadly signified by an increase in free time, opportunities to maximize the utility of one's free time, and enjoyment of leisure activities that are socially structured (24). The aging activity theory postulates that older people who maintain active social activities have a higher quality of life in later life and a more successful aging process than those who do not (25, 26). Active lifestyle arrangements among older people highlight the potential of leisure activities in improving subjective health in later life (4, 10, 27, 28).

Although previous studies have analyzed the impact of a leisure and entertainment lifestyle (LEL) on health, they are limited to the role of certain types of LEL or focus on only a single dimension of health; moreover, rare studies focus on older people living alone in China (29–31). Leisure activity has been proven to moderate the relationship between living alone and poor physical health, mental health, and social health among Japanese, Korean, Singaporean, and American, older adults living alone (32–34). Therefore, based on the previous literature mentioned above, we proposed the hypothesis that LEL significantly improves the multidimensional health of older people living alone in China.

How does LEL affect the multidimensional health of older people living alone? Some scholars pointed out that leisure and entertainment life are a means to improve the efficacy of physical exercise, and effective exercise can improve health promotion (35–37). Studies have shown that a rich life of leisure and social interaction increases positive life attitudes such as happiness and enthusiasm for life, relieves loneliness, and increases subjective health perception (38–40). From the perspective of social capital, active leisure and entertainment life would enhance personal social trust and social adaptability (41, 42). This means that leisure and entertainment life can positively affect the health of older adults living alone through exercise efficacy, positive life attitudes, and social trust. However, these hypotheses have not been explored fully in the currently limited literature, especially in China.

To sum up, this study explores the effects of LEL on the health of older people living alone from the following aspects. First, ordinary least square regression (OLS) was adopted to analyze the influence of LEL on multidimensional health. However, the limited studies on this topic identify critical challenges in terms of the potential for residual confounding due to omitted variables, differential measurement error, or a possible causal relationship between LEL and multidimensional health. We should further address the endogenous problem. Hence, under considering endogeneity, we adopted a simultaneous equations model consisting of OLS and two-stage least squares (2SLS), as well as an instrumental variable introduced in the 2SLS model to correct the results. This will help to more robustly estimate the effect of LEL on the multidimensional health of older people living alone. Finally, we adopted a mediating effect model to explore the mechanism of this effect.

## Methods

### Study design

We obtained data from the Chinese General Social Survey 2017 (CGSS). The CGSS is a national, comprehensive, and continuous academic survey project for Chinese residents conducted by the China Survey and Data Center of Renmin University of China. These data are representative of the scientific research value recognized by academia at present. The survey adopted a multi-stage stratified sampling method, collecting 12,582 representative valid sample data from 125 counties in 31 province-level regions of mainland China (43).

The 2017 CGSS data include comprehensive information on the population and social attributes, health, family, work, leisure and entertainment life, and social attitudes of Chinese residents of all age groups over 18 years of age. Following the purpose of our research, we filtered the age variable as “over 60 years old” and the residence status as “live alone.” Finally, 865 samples were included in the analysis after deleting the missing values and outliers of the sample.

### Variables and measures

#### Multidimensional health

According to the World Health Organization, health is a state of physical, mental, and social well-being (44). Health is dynamic, multidimensional, and continuous. Some scholars regard health as a state of personal experience and reflect health status through subjective health evaluation indicators (45, 46). The physical health spectrum can be categorized as ranging from a minimum state defined by an inability to work or care for personal needs, to an optimal state expressed by no complaints and a high level of energy (47, 48). This means that the level of physical health is related to the degree of physical function and activity limitation brought about by diseases (49). Mental health includes positive emotions and positive functioning, and the measurement of negative or positive emotions can reflect an individual's mental health status (50, 51). Social health is defined in terms of social participation in activities and interpersonal interactions (52). The dimensions we choose for the multidimensional health of older people living alone included physical health, mental health, and social health. According to previous research, multidimensional health was measured by three questions in the CGSS 2017: “How often has your work or daily activities been affected by health problems?” “How often did you feel depressed or depressed?,” and “How often did you participate in social activities in your free time?,” respectively (3, 53, 54). A five-point Likert scale was used to evaluate the levels of each dimension. The higher the score, the better the health status.

#### Leisure and entertainment life (LEL)

LEL was measured by the question “The frequency of engaging in the following activities in your free time.” The answers included watching television, watching movies, shopping, reading, watching performances, gathering with relatives, gathering with friends, listening to music, physical exercise, watching sports, doing crafts, and surfing the Internet. The answers to each question ranged from 1 to 5. 1 = never, 2 = a few times a year, 3 = a few times a month, 4 = a few times a week, and 5 = every day. The LEL index was calculated by adding up the scores of all the responses. A higher score indicated a higher level of leisure and entertainment. The Cronbach's α of LEL was 0.812, and the KOM value was 0.701.

#### Mediating variables

Exercise efficacy, positive life attitudes, and social trust as mediating variables were further examined as potential mechanisms by which LEL affects multidimensional health in the mediating effect model. A large body of evidence suggested that a physically active lifestyle is associated with a reduced risk of chronic disease, all-cause mortality, and cognitive health (55). Public health recommendations in many countries emphasize regular participation in moderate-intensity physical activity (at least 5 days per week, 30 min or more per day) (56). Some studies asked respondents to report activities that “make you sweat at least” to measure exercise efficacy by combining light-intensity physical activity and moderate-to-vigorous physical activity for this domain across different ages and genders (57–59). This measure of exercise efficacy is also applicable in Chinese health life study, and it is expressed as “the frequency of physical activity that lasts more than 30 min and makes you sweat” in CGSS 2017.

Happiness is commonly understood as how much one likes one's life or, more formally, the degree to which one positively evaluates one's life as a whole. A central element in the definition is subjective “evaluation” or “liking” of life. The set-point theory considers evaluation as a stable attitude toward life. Happiness is also considered a happy disposition and positive attitude toward life (60, 61). This definition of happiness as an attitude toward life stresses the consistency in affective response, while others see it as a belief system, especially for older people (40, 62). Therefore, we used the evaluation of happiness of older people living alone as their positive attitude toward life, which is evaluated by the “degree of happiness in life” in CGSS 2017.

Social trust refers to trust in people in general, and it is a widely used social capital variable. Standard trust is measured by the share of a population that answers that most people can be trusted when asked the question “In general, do you think most people can be trusted?” (63, 64). Although this question has been criticized for being vague and difficult to answer without specific knowledge of whom to trust, it is acceptable because social trust is in essence trust without specific information (65). Likewise, social trust is expressed by the “degree of trust in others in society” in CGSS 2017, which is easier for Chinese people to understand (66).

The answers for three variables were divided into five grades with 1–5 points. A higher score indicated a higher degree of exercise efficacy, positive life attitude, and social trust of the survey respondents.

#### Control variables

Some studies indicated that sociodemographic characteristics and socioeconomic status would influence the health of older people (13, 28, 42, 67). With reference to previous studies, the following variables were considered: household registration, gender, age, education level, marital status, work status, the number of children, socioeconomic status (1 = well below average, 2 = below average, 3 = average, 4= above average, and 5 = well above average), and cognitive ability (1 = very poor, 2 = relatively poor, 3 = ordinary, 4 = better, and 5 = very good). Table 1 shows the details of the settings and measurements of the above variables.

**Table 1:** OLS estimation of the correlation of LEL and multidimensional health (*N* = 865).

**Variables**	**Model 1 physical health**			**Model 2 mental health**			**Model 3 social health**		
	**β**	**95% CI**		**β**	**95% CI**		**β**	**95% CI**	
LEL	0.042***	0.028	0.055	0.027***	0.012	0.042	0.054***	0.038	0.070
	(0.007)			(0.007)			(0.008)		
Sex (ref: female)	0.215***	0.061	0.369	0.133*	−0.011	0.277	−0.174**	−0.331	−0.017
	(0.079)			(0.073)			(0.080)		
Age	0.002	−0.009	0.012	0.012**	0.002	0.022	0.006	−0.005	0.018
	(0.005)			(0.005)			(0.006)		
Household registration (ref: Urban)	−0.322***	−0.535	−0.109	−0.131	−0.347	0.085	0.590***	0.383	0.798
	(0.108)			(0.110)			(0.106)		
Marital status (ref: married)	0.017	−0.163	0.196	0.025	−0.151	0.202	0.056	−0.141	0.253
	(0.091)			(0.090)			(0.100)		
Education	−0.035	−0.123	0.052	−0.019	−0.105	0.067	−0.150***	−0.240	−0.059
	(0.044)			(0.044)			(0.046)		
Working status (ref: no)	0.314***	0.129	0.498	0.031	−0.141	0.202	−0.111	−0.305	0.084
	(0.094)			(0.087)			(0.099)		
Number of children	−0.015	−0.053	0.023	−0.021	−0.061	0.018	−0.032	−0.070	0.006
	(0.019)			(0.020)			(0.019)		
Socioeconomic status	0.127***	0.031	0.223	0.160***	0.080	0.240	0.162***	0.072	0.253
	(0.049)			(0.041)			(0.046)		
Hospital experience (ref: not hospitalized)	−0.837***	−0.999	−0.675	−0.285***	−0.438	−0.132	−0.113	−0.276	0.050
	(0.083)			(0.078)			(0.083)		
Cognitive ability	0.061	−0.021	0.142	0.073**	0.001	0.144	0.041	−0.038	0.121
	(0.042)			(0.037)			(0.040)		
Intercept	2.228***	1.273	3.184	1.720***	0.760	2.68	0.754	−0.242	1.749
	(0.487)			(0.489)			(0.507)		
*N*	865			865			865		
*F*	32.15***			11.94***			9.18***		
*R* ^2^	0.257			0.127			0.104		

Robust standard errors in parentheses. *P < 0.1, **P < 0.05, and ***P < 0.01.

### Analytical strategy

Descriptive statistics were used to present the means and standard deviations of the main study variables in the model. Variance inflation factor (VIF) was used as the multicollinearity diagnosis index for variables, VIF < 3.

The relationship between LEL and multidimensional health was established based on a simultaneous equation. First, the OLS model was used to estimate the correlation between the three outcome healthy indicators and all explanatory variables of older people living alone. The model's equation can be set as follows:


(1) $$\begin{array}{l} {\text{Y}}_{i}=\beta_{0}+\beta_{1}{\text{L}}_{i}+\delta {\text{X}}_{i}+\varepsilon_{i} \end{array}$$

where Y_*i*_ is the health level (physical health, mental health, and social health), L_*i*_ is the LEL, X_*i*_ is the control variable, and β_1_ is the estimated parameter reflecting the effect of LEL on the health status of older people living alone.

Second, we considered the empirical results caused by the yield biased from the OLS model. Therefore, we also used the average score of the LEL of the older people living alone at the provincial level (*LELm*) as an instrumental variable to solve the endogeneity problem. Furthermore, the 2SLS model, presented in equations (2) and (3), was adopted to estimate the result. Similar strategies were adopted in other studies (45, 68, 69).


(2) $$\begin{array}{l} {\text{The first stage}:\text{ L}}_{i}=\alpha_{0}+\alpha_{1}{\text{Lm}}_{i}+\delta {\text{X}}_{i}+v_{i} \end{array}$$

(3) $$\text{The second stage}:{\text{Y}}_{i}={\text{λ}}_{0}+{\text{λ}}_{1}{\text{L}}^{\wedge }{}_{i}+{\text{δX}}_{i}+{\text{ε}}_{\text{i}}$$

Equation (2) is the estimation equation of the first stage, where Lm_*i*_ represents the selected instrumental variable *LELm*, α_1_ is the estimated parameter, and *v*_*i*_ is the linear random error. Equation (3) is the estimation equation of the second stage, where L^∧^_*i*_ is the predicted value of L_*i*_ in equation (2), and λ_1_ is the estimated parameter that represented the predictive effect of the first-stage regression result. The other variables have the same meanings as in equation (1).

Finally, we set up the mediating effect model to explore the mechanism of LEL affecting multidimensional health (54, 70). The equation used is as follows:


(4) $$\begin{array}{lll} {\text{Y}}_{i} & = & \alpha {\text{L}}_{i}+\delta {\text{X}}_{i}+\varepsilon_{\text{i}} \end{array}$$

(5) $$\begin{array}{lll} {\text{M}}_{i} & = & \theta {\text{L}}_{i}+\delta {\text{X}}_{i}+\mu_{\text{i}} \end{array}$$

(6) $$\begin{array}{lll} {\text{Y}}_{i} & = & \alpha^{\prime }{\text{L}}_{i}+\gamma {\text{M}}_{i}+\delta {\text{X}}_{i}+\nu_{\text{i}} \end{array}$$

where α is the total effect of L_*i*_ (LEL) on Y_*i*_ (multidimensional health), θ is the effect of LEL on mediating variable M_*i*_ (exercise efficacy, positive life attitude, and social trust), α′ is the direct effect of L_*i*_ on health after controlling for mediating variable M_*i*_, and γ is the effect of mediating variable M_*i*_ on Y_*i*_. The ratio of indirect effect to total effect is calculated as θγ/α.

Model estimates may be inconsistent due to sample bias or causal relationships between variables; therefore, we used the substitution variable and model for the robustness test. We used the total score of the respondents' answers to the frequency of using media tools and entertainment with relatives and friends in the CGSS 2017 as a substitution variable (*LELs*) for LEL, and self-rated general health level as a substitution variable for multidimensional health. Some studies showed that the estimated effects of using OLS and the ordered probit model are virtually identical; therefore, we also adopted the ordered probit model to test the robustness of the OLS estimation results (68, 71, 72). Detailed test results are provided in Supplementary Table S1. All data were analyzed using Stata version 17.0.

## Result

### Descriptive statistics

**Figure 1:**
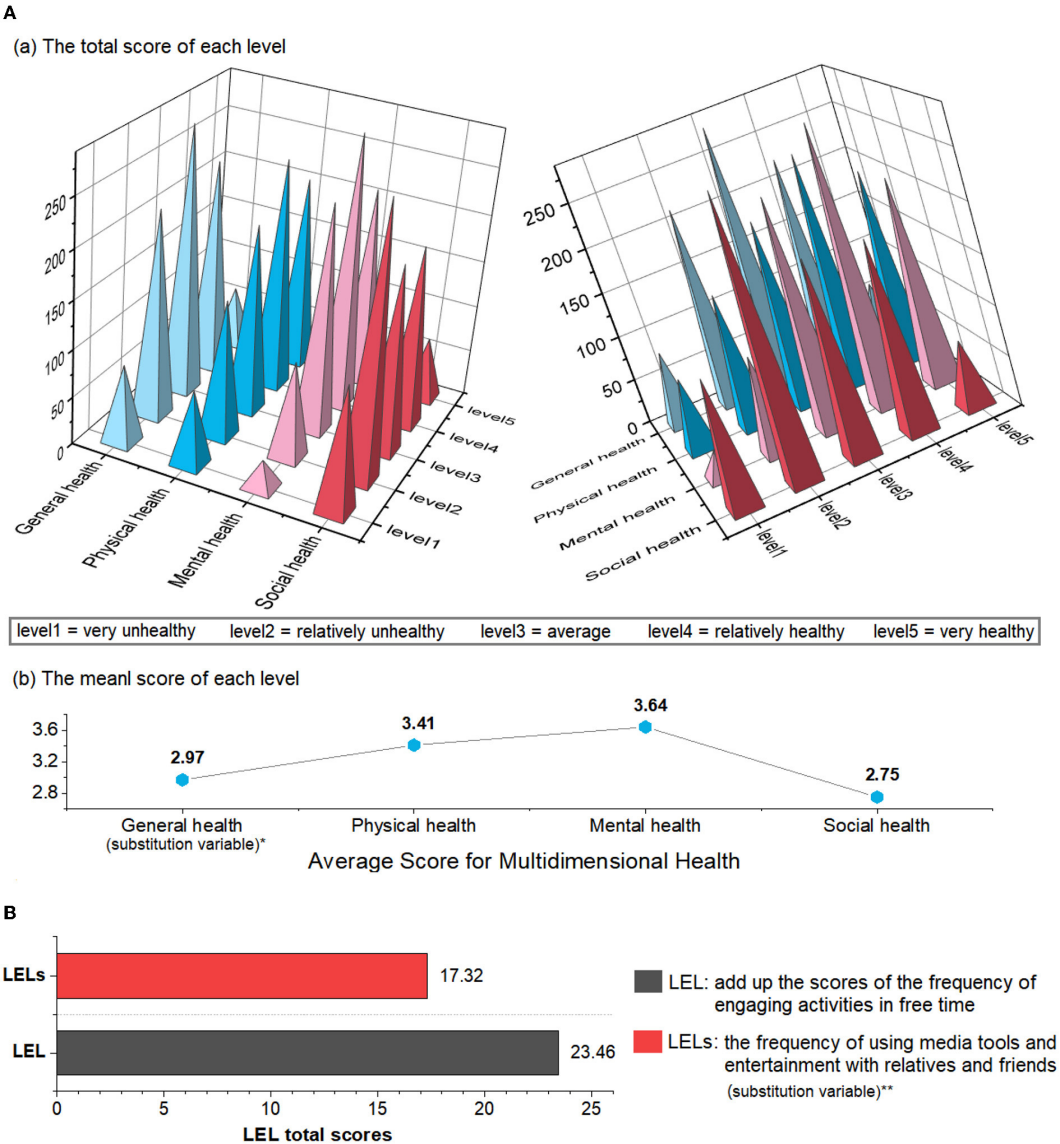
**(A)** Multi-dimensional health level and **(B)** leisure and entertainment life (LEL) level of older people living alone in China. *General health level as substitution variable for multi-dimensional health is examined for robustness check of the model robustness test. **The frequency of using media tools and entertainment with relatives and friends as substitution variable for leisure and entertainment life is examined for robustness check of the model robustness test.

Figure 1 shows the levels of multidimensional health and the LEL index. In terms of physical health, 28.21% of the older people living alone thought they were relatively healthy, and 23.58% were very healthy. Regarding mental health, 33.18% of the older people living alone reported being relatively healthy, and 24.39% reported they were very healthy. Regarding social health, 22.08% of the older people living alone reported they were relatively healthy, and only 7.63% were very healthy. The study calculated the average level of health in each dimension: Physical health score was 3.41 ± 1.26, mental health score was 3.64 ± 1.07, and social health score was 2.75 ± 1.18. Overall, older people living alone in China rated mental health the highest, followed by physical health and social health. Moreover, the LEL index of older people living alone was 23.46 ± 6.53.

**Figure 2:**
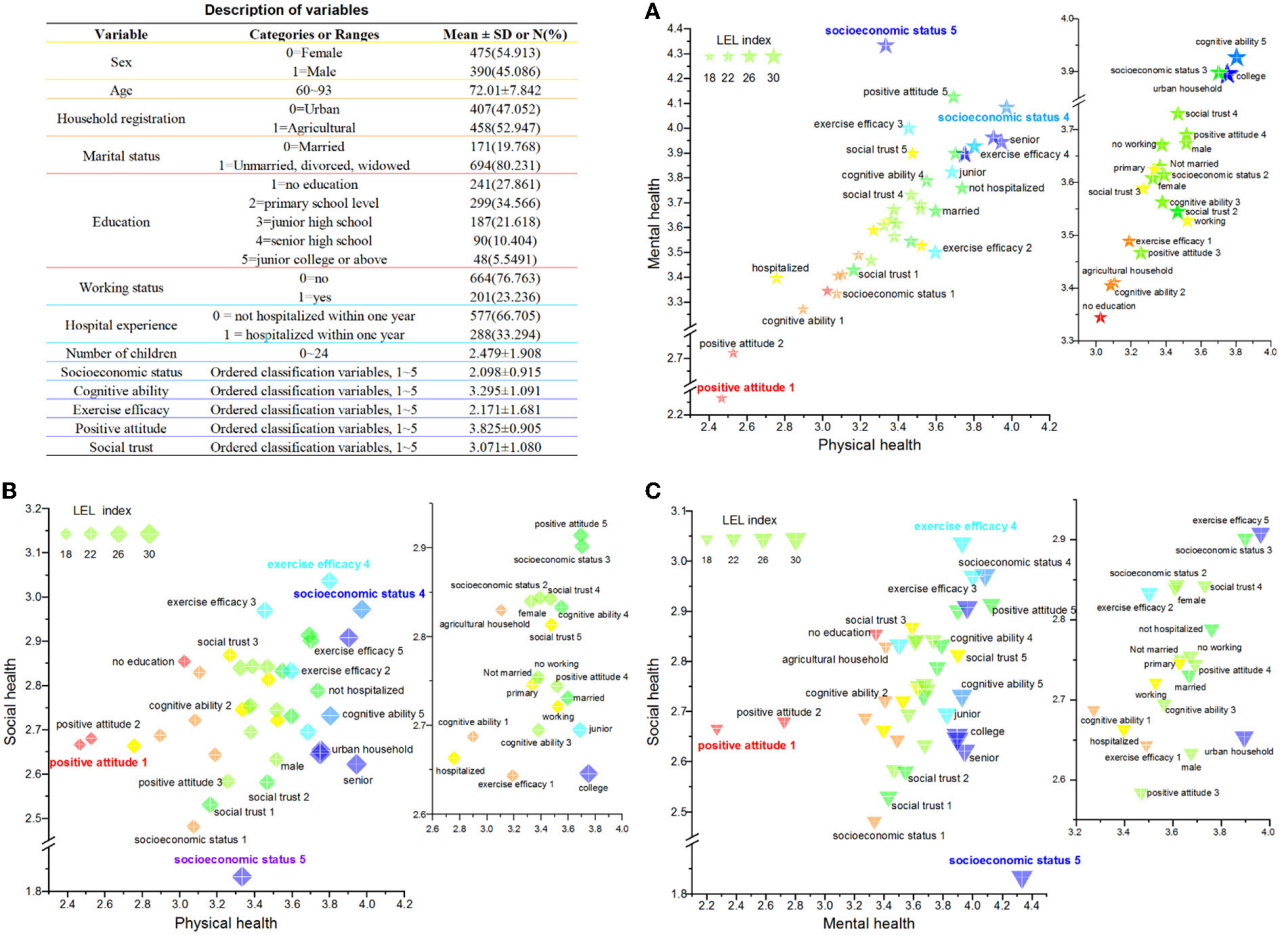
Variable distribution differences in multi-dimensional health and leisure and entertainment life (LEL) of older people living alone in China. **(A)** Variable distribution differences in physical health—mental health—LEL. **(B)** Variable distribution differences in physical health—social health—LEL. **(C)** Variable distribution differences in mental health—social health—LEL.

Detailed information on other explanatory indicators is shown in Figure 2. Among the 865 respondents, the average age was 72.01, and 54.91% of respondents were women. The proportion of agricultural household registrations was 52.95%. Furthermore, 79% of the overall sample were non-marital status (unmarried, divorced, or widowed). Finally, they had 2.48 children on average.

### Results of the OLS model

Table 1 and Figure 2 show the OLS estimation correlations between the explanatory variables and the multidimensional health of the older people living alone. LEL significantly improved the social health of older people living alone (β = 0.054, *P* < 0.01), followed by physical health (β = 0.042, *P* < 0.01) and mental health (β = 0.027, *P* < 0.01).

Among sociodemographic characteristics, older men living alone had a higher level of physical health than women (β = 0.215, *P* < 0.01), but the opposite effect was observed in predicting social health (β = −0.174, *P* < 0.05). Older people living alone with agricultural household registration had a lower level of physical health than those with urban household registration (β = −0.322, *P* < 0.01), but the opposite effect was observed in predicting social health (β = 0.590, *P* < 0.01). Interestingly, the level of mental health has improved significantly with age, and there was no significant effect on physical health and social health. However, there was a significant negative correlation between education level and social health, which means that the level of social health declined as education level increased.

In addition, according to the current state of older people living alone, those who still worked had better physical health (β = 0.314, *P* < 0.01). Old people who were not hospitalized had better physical and mental health than those who were hospitalized within the past 1 year (β_physicalhealth_ = −0.837, β_mentalhealth_ = −0.285, *P* < 0.01). The cognitive ability of older people was significantly positively correlated with their mental health (β =0.073, *P* < 0.05). Moreover, socioeconomic status was positively correlated with the three dimensions of health ((β_physicalhealth_ = 0.127, β_mentalhealth_ = 0.160, β_socialhealth_ = 0.162, *P* < 0.01).

### Results of the 2SLS model

The OLS model confirmed significant correlations between LEL and the three dimensions of health in older people living alone, but any observed causal relationship must be interpreted with caution. We used the LEL's average scores of the older people at the provincial level as instrumental variables and conducted 2SLS estimation for endogeneity. To further confirm the validity of the instrumental variable, a weak instrumental variable test was needed before the estimation of the 2SLS model. According to Angrist and Pischke, when the F-value of the minimum eigenvalue statistic is > 10, the instrumental variables are not weak (73). After the weak instrumental variable test, the minimum eigenvalue statistic was 80.30, which was far > 10, and the partial R-squared was 0.086, suggesting there was no problem of weak instruments. The relevance assumption was thus satisfied, and we can use the *LELm* as an instrumental variable, as it was not part of the error term of the second-stage regression.

According to Table 2, the empirical results of the 2SLS confirmed that the active LEL of the older people living alone had a positive impact on their health, which was similar to the OLS model. Except for social health, the regression coefficients of LEL had a significant impact on physical health (β = 0.098, *P* < 0.05) and mental health (β = 0.051, *P* < 0.05). The high regression coefficient of LEL indicated that, without introducing the instrumental variable, OLS underestimated the impact of the LEL of older people living alone on their physical health and mental health.

**Table 2:** 2SLS estimation of the effect of LEL on multidimensional health.

**Variables**	**1st stage LEL**			**2nd stage Physical health**			**2nd stage Mental health**			**2nd stage Social health**		
	**β**	**95% CI**		**β**	**95% CI**		**β**	**95% CI**		**β**	**95% CI**	
LELm	0.543***	0.410	0.675									
	(0.0606)											
LEL				0.098***	0.048	0.149	0.051**	0.004	0.097	0.001	−0.051	0.053
				(0.026)			(0.024)			(0.027)		
Other variables	Controlled			Controlled			Controlled			Controlled		
Intercept	13.94***	8.935	18.944	0.740	−0.877	2.357	1.099	−0.409	2.607	2.131**	0.445	3.816
	(2.441)			(0.825)			(0.769)			(0.860)		
*N*	865			865			865			865		
*R* ^2^	0.451			0.204			0.114			0.053		
Durbin chi2				5.749**			1.172			4.633**		
*F* /Wu–Hausman *F*	68.20***			5.700**			1.156			4.588**		

Robust standard errors in parentheses. *P < 0.1, **P < 0.05, and ***P < 0.01.

### How LEL affects multidimensional health of older people living alone

Table 3 shows the mediating effects of exercise efficacy, positive life attitudes, and social trust. According to equation (5), the results showed that LEL and exercise efficacy were positively correlated, with a regression coefficient of 0.122 at the 0.01 level, as shown in column (1) of Table 3. When LEL and exercise efficacy were both included in equation (6), the direct impact of LEL on physical health was 0.033 and significant at the 0.01 level, as shown in column (2) of Table 3. Meanwhile, the regression coefficient of exercise efficacy was 0.068 and significant at the 0.01 level. Combining the results of the total effect (coefficient = 0.042, *P* < 0.01) of LEL on physical health in Table 1, exercise efficacy was considered to play a partial mediating role between LEL and physical health; the ratio of the intermediary effect in the total effect was 19.75%. Similarly, exercise efficacy was considered to play a partial mediating role between LEL and mental health; the ratio of the intermediary effect to the total effect was 24.85%. A positive life attitude was considered to play a partial mediating role between LEL and physical health, and LEL and mental health; the ratio of the intermediary effect to the total effect was 10.65and 26.83%, respectively. However, according to the mediation effect test procedure, after the bootstrap test (5000 samples), the indirect effect of exercise efficacy was not significant between LEL and social health, so did the indirect effect of positive life attitudes was not significant between LEL and social health. Likewise, the indirect effect of social trust was not significant, as no mediating role of social trust was found between LEL and multidimensional health.

**Table 3:** Results of the mediation test.

**Variables**	**(1) Exercise efficacy**			**(2) Physical health**			**(3) Mental health**			**(4) Social health**		
	**β**	**95% CI**		**β**	**95% CI**		**β**	**95% CI**		**β**	**95% CI**	
LEL	0.122***	0.99	0.145	0.033***	0.19	0.48	0.020**	0.04	0.36	0.050***	0.33	0.67
	(0.012)			(0.007)			(0.008)			(0.009)		
Exercise efficacy				0.068***	0.19	0.117	0.055**	0.08	0.102	0.035	−0.20	0.90
				(0.025)			(0.024)			(0.028)		
Other variables	Controlled			Controlled			Controlled			Controlled		
Intercept	0.282	−10.44	1.608	2.209***	1.262	3.157	1.705***	0.749	2.66	0.744	−0.249	1.736
	(0.676)			(0.483)			(0.487)			(0.506)		
*N*	865			865			865			865		
*R* ^2^	0.258			0.263			0.132			0.106		
**Variables**	**(5) Positive life attitude**			**(6) Physical health**			**(7) Mental health**			**(8) Social health**		
	β	**95% CI**		β	**95% CI**		β	**95% CI**		β	**95% CI**	
LEL	0.021***	0.11	0.32	0.037***	0.23	0.51	0.020***	0.05	0.34	0.054***	0.38	0.70
	(0.005)			(0.007)			(0.007)			(0.008)		
Positive life attitude				0.213***	0.122	0.304	0.345***	0.259	0.431	−0.005	−0.099	0.089
				(0.046)			(0.044)			(0.048)		
Other variables	Controlled			Controlled			Controlled			Controlled		
Intercept	1.750***	10.16	2.483	1.856***	0.901	2.81	1.116**	0.170	20.62	0.762	−0.247	1.772
	(0.374)			(0.486)			(0.482)			(0.514)		
*N*	865			865			865			865		
*R* ^2^	0.161			0.277			0.198			0.104		
**Variables**	**(9) Social trust**			**(10) Physical health**			**(11) Mental health**			**(12) Social health**		
	β	**95% CI**		β	**95% CI**		β	**95% CI**		β	**95% CI**	
LEL	−0.005	−0.019	0.09	0.042***	0.28	0.55	0.027***	0.13	0.42	0.054***	0.39	0.70
	(0.007)			(0.007)			(0.008)			(0.008)		
Social trust				0.063*	−0.009	0.136	0.112***	0.046	0.179	0.081**	0.008	0.154
				(0.037)			(0.034)			(0.037)		
Other variables	Controlled			Controlled			Controlled			Controlled		
Intercept	2.454***	1.480	3.429	20.73***	1.107	30.38	1.444***	0.473	2.416	0.554	−0.439	1.548
	(0.497)			(0.492)			(0.495)			(0.506)		
*N*	865			865			865			865		
*R* ^2^	0.029			0.260			0.139			0.109		

Robust standard errors in parentheses. *P < 0.1, **P < 0.05, and ***P < 0.01.

## Discussion

Based on the theory and results from the OLS model, active LEL is significantly associated with better health for older people living alone. Using the simultaneous OLS and 2SLS equation models, we obtained unbiased estimators of the outcome. After endogeneity and robustness tests, we found that more active LEL was associated with 0.098% higher physical health and 0.051% higher mental health; the OLS results may have underestimated the LEL's positive effect. Our positive results were similar to those found in other studies that did not consider endogeneity (74–76). The results robustly confirmed that LEL can promote the physical health and mental health of older people living alone. Furthermore, through mediation analysis, we confirmed a partial mediating effect of exercise efficacy between LEL and physical and mental health, and a similar mediating effect of active life attitudes between LEL and physical and mental health.

According to the disengagement theory and activity theory state, the majority of older people are not willing to withdraw from the social systems to which they belong (77, 78). Disengagement theory argues that as an inevitable disengagement process, aging is associated with social isolation and reduced social participation (79). Retirement, the departure of children, and the beloved gone gradually shape the “passive state” of older people living alone. With the separation from the original work roles and family roles, there is a loss of role function in older people, resulting in their physical and mental imbalances (80). Older people living alone experience greater social isolation if they withdraw from social structure. Successful aging is a dynamic process of transition from passive aging to active aging. Therefore, as a representative view of successful aging, activity theory claims that social activities with alternative compensation functions are necessary to achieve successful aging (79, 81). Activity theory argues against sedentary lifestyles, arguing that maintaining active physical and social activity in older adults is critical to their health and well-being (82). Older people living alone retain psychological and social needs similar to younger adults, and their way to resist social withdrawal is to stay socially active in leisure and entertainment to enhance self-adaptation and social integration. Older people living alone gain more advantages by engaging in leisure time because they may not have family obligations such as caring for a spouse or grandchildren compared with older people living together. Environmental infrastructure support in public settings, such as seniors' outdoor exercise venues (playgrounds and exercise parks) and chess rooms in the community, provides opportunities for physical activity and social connectedness for older people. Except for traditional media, the Internet and smart media provide broader information environment support to promote the activeness of older people living alone. In recent years, there has been a boom in short videos and live broadcasts, such as on square dancing, singing, and physical exercise, on the Internet in China, which may promote positive life attitudes and motivation to exercise effectively among seniors living alone. Leisure styles, such as sports participation and watching of performances and games, may promote positive emotional states and enhance social interaction of them.

There are several possible explanations for the lack of a relationship between leisure and entertainment and the social health of older people living alone. One explanation is that the number and structure of recreational activities among older people living alone, such as home activities and outdoor activities, may offset one another's effects on social health (75, 83, 84). In the context of rapid urbanization and modernization in China, as people gradually move to different regions, the relatives and neighbors of the older people living alone will become estranged due to geographic locations, and members of the new community are also relatively unfamiliar. At present, the older generation in China can communicate with the outside world through mobile phones and the Internet, which may reduce the intimate association between leisure, entertainment, and social health. Therefore, the LEL of older people living alone may increase the possibility of social interaction, but there are still challenges in prompting them to actively improve their social health premised on social trust or social reciprocity in a short period (85).

This study measured the multidimensional health differences among older people living alone at the sociodemographic level. Older women were shown to have poorer physical health; this is likely because of women's vulnerability due to a higher risk of chronic illnesses stemming from longer life expectancy and less access to appropriate healthcare (2, 86–88). Nevertheless, older Chinese women may have active social participation because they are more likely to chat, share, shop, and participate in square dancing (88–91). Another interesting finding is that older people living alone had better mental health as their age increased. One possible explanation is that older individuals living alone may have lower expectations from life as they age (92).

Compared with urban older people, older people living alone in rural areas reported lower physical health but higher social health. A similar result about lower physical health was reported in previous studies (93, 94). Limited economic resources, backward infrastructure, and limited access to health information all hinder health-promoting activities among older people living in rural areas (29). However, neighbors in rural China are closer, especially the older generation, as they are affected by the clan system and blood ties. Thus, they have more opportunities and willingness to participate in human relations activities in villages (95, 96). A further interesting finding is that education was significantly negatively associated with social health, which is the opposite of other studies showing that older people with higher education are significantly more likely to participate in social activities (97, 98). This may be because older people with high education living alone are more dependent on new media for information than on social participation (99).

These findings have important implications in making active aging policies to ensure the well-being of older people living alone. First, installing and supporting age-friendly and suitable outdoor exercise equipment are necessary health promotion measures in the location and settings where seniors live alone, especially for vulnerable seniors living alone in Chinese rural communities, with multiple diseases and low incomes. The two-way linkage between online sharing on the Internet and offline leisure activities is a way of health promotion that keeps pace with the times against the background of the popularization of network media. Propagating health information through new media, such as the Internet, WeChat, Douyin, and other mobile apps, can help expand the life cycle of older people living alone.

## Conclusion

Health problems of older people living alone in China have aroused widespread concern in the society. We explored the relationship and mechanism between LEL and multidimensional health of older people living alone in China for the first time using simultaneous equations. We obtained unbiased estimators of the significant positive outcome between LEL and the multidimensional health of older people living alone by using the model of OLS and 2SLS models. We robustly confirmed that active LEL can improve the multidimensional health of older people living alone. Furthermore, a mediation analysis indicated partial mediating effects of exercise efficacy between LEL-physical health and LEL-mental health, among others, on the effects of active life attitudes between LEL-physical health and LEL-mental health. These findings have important implications in making active aging policies for the well-being of older people living alone.

## Limitation

Our results must be considered with caution, due to some study limitations that must be acknowledged. First, although our study sample may be fairly representative of the older population living alone in China and our research comprises a cross-sectional, relevant survey, we cannot rule out the possibility of reverse causation. Hence, future prospective studies are necessary (100). Furthermore, even though referring to the previous research experience, there is still a little limitation when adopting the question in CGSS2017 to measure physical health that the participant might consider mental issues as a health problem impacting on work or daily activities and answer accordingly. In addition, health outcomes of older adults living alone are not only affected by their short-term leisure lifestyle in later life, but also by a combination of factors such as the long-term living environment, social welfare, early life experiences, and household spending (101–106). Due to the selection of sample in this study, we did not conduct an in-depth analysis of these factors, and the living arrangements of older people living alone during different survey follow-up periods need to be explored in future research (10). Finally, although this study used the latest 2017 CGSS data, considering the past 2 years, COVID-19 might have an impact on the leisure lifestyles of the older population. Thus, it is necessary to follow up with the latest data and fully consider the relationship between the lifestyle and health status of older people living alone during the pandemic (28).

## Data availability statement

The datasets used and analyzed during the current study are available from the corresponding author on reasonable request.

## Author contributions

QHW was responsible for the overall design of the research and revised the paper. YHQ conducted, analyzed the results, and drafted the manuscript. SCJ, PFG, and JCL substantially contributed to data acquisition. RZW and XYQ assisted with the literature review. JJL contributed to the interpretation of results and in writing the manuscript. All authors contributed to this manuscript. All authors approved of the current version of this manuscript for publication.

## Funding

This study was supported by the National Key Social Science Fund of China (Grant No.19AZD013).

## Conflict of interest

The authors declare that the research was conducted in the absence of any commercial or financial relationships that could be construed as a potential conflict of interest.

## Publisher's note

All claims expressed in this article are solely those of the authors and do not necessarily represent those of their affiliated organizations, or those of the publisher, the editors and the reviewers. Any product that may be evaluated in this article, or claim that may be made by its manufacturer, is not guaranteed or endorsed by the publisher.
