# Supplementary material for: Can leisure and entertainment lifestyle promote health among older people living alone in China?—A simultaneous equation approach
Source: Front Public Health. 2022 Sep 29;10:967170. doi: 10.3389/fpubh.2022.967170 (PMC9558104; doi:10.3389/fpubh.2022.967170)
Supplement: Supplementary file 1 [file Table_1.docx]

1. **Robust test**

In this part, we used the substitution variable (general health) of multi-dimensional health, substitution variable (LELs) of LEL, and Order probit model to test the robustness of the foregoing results. According to the second and third columns of Table S1, LEL has a positive effect on the self-reported health of the elderly living alone, and the results of OLS and 2SLS are consistent with the results of Table 1 and Table 2 in the main text in the degree and direction of change. LEL all had positive effects on physical, mental, and social health after prediction using an ordered probit model, which is consistent with the previous OLS results. Further, after LELs were replaced by LELs, we performed OLS, 2SLS, and mediation analyses again, and their results were consistent in direction and significance with those in Tables 1, 2, and 3 in the main text. Therefore, we can conclude that the leisure and entertainment life have a significant promote effect on the physical, mental and social health of the elderly living alone. This result is robust.

**Table S1. Results of robust test.**

| **Variables** | **Multi-health proxy: General health** | | **Order probit** | | | **OLS** | | | **2SLS** | | |
| --- | --- | --- | --- | --- | --- | --- | --- | --- | --- | --- | --- |
|  | **OLS** | **2SLS** | **Physical health** | **Mental health** | **Social health** | **Physical health** | **Mental health** | **Social health** | **Physical health** | **Mental health** | **Social health** |
| LEL | 0.024^***^ | 0.075*** | 0.042*** | 0.029*** | 0.051*** |  |  |  |  |  |  |
|  | (0.007) | (0.023) | (0.007) | (0.008) | (0.008) |  |  |  |  |  |  |
| LEL proxy: LELs |  |  |  |  |  | 0.035^***^ | 0.031^***^ | 0.137^***^ | 0.169*** | 0.084** | 0.051 |
|  |  |  |  |  |  | (0.010) | (0.009) | (0.009) | (0.049) | (0.043) | (0.044) |
| Other variables | Controlled | Controlled | Controlled | Controlled | Controlled | Controlled | Controlled | Controlled | Controlled | Controlled | Controlled |
| *N* | 865 | 865 | 865 | 865 | 865 | 865 | 865 | 865 | 865 | 865 | 865 |
| *R2/ Pseudo-R*^2^ | 0.191 | 0.134 | 0.094 | 0.048 | 0.037 | 0.24 | 0.122 | 0.238 | 0.079 | 0.089 | 0.164 |

Robust standard errors in parentheses. * p < 0.1, ** p < 0.05, *** p < 0.01. Estimates of cut-off omitted due to space limitation.

**Table S1. Results of robust test. (continued)**

| Variables | (1) | (2) | (3) | (4) | (5) | (6 | (7) | (8) | (9) | (10) | (11) | (12) |
| --- | --- | --- | --- | --- | --- | --- | --- | --- | --- | --- | --- | --- |
|  | Exercise efficacy | Physical health | Mental health | Social health | Positive life attitude | Physical health | Mental health | Social health | Social trust | Physical health | Mental health | Social health |
| LEL proxy: LELs | 0.090^***^ | 0.026^***^ | 0.025^***^ | 0.134^***^ | 0.028^***^ | 0.028^***^ | 0.021^**^ | 0.138^***^ | 0.002 | 0.035^***^ | 0.031^***^ | 0.136^***^ |
|  | (0.015) | (0.010) | (0.010) | (0.010) | (0.007) | (0.010) | (0.009) | (0.010) | (0.010) | (0.010) | (0.009) | (0.009) |
| Exercise efficacy |  | 0.097^***^ | 0.068^***^ | 0.030 |  |  |  |  |  |  |  |  |
|  |  | (0.024) | (0.022) | (0.024) |  |  |  |  |  |  |  |  |
| Positive life attitude |  |  |  |  |  | 0.226^***^ | 0.349^***^ | -0.040 |  |  |  |  |
|  |  |  |  |  |  | (0.047) | (0.044) | (0.043) |  |  |  |  |
| Social trust |  |  |  |  |  |  |  |  |  | 0.058 | 0.108^***^ | 0.072^**^ |
|  |  |  |  |  |  |  |  |  |  | (0.037) | (0.034) | (0.035) |
| Other variables | Controlled | Controlled | Controlled | Controlled | Controlled | Controlled | Controlled | Controlled | Controlled | Controlled | Controlled | Controlled |
| _cons | 2.132^***^ | 2.589^***^ | 1.814^***^ | 0.059 | 1.885^***^ | 2.371^***^ | 1.301^***^ | 0.199 | 2.297^***^ | 2.663^***^ | 1.709^***^ | -0.042 |
|  | (0.694) | (0.490) | (0.477) | (0.451) | (0.371) | (0.493) | (0.466) | (0.461) | (0.485) | (0.500) | (0.486) | (0.457) |
| *N* | 865 | 865 | 865 | 865 | 865 | 865 | 865 | 865 | 865 | 865 | 865 | 865 |
| *R*^2^ | 0.163 | 0.254 | 0.132 | 0.239 | 0.160 | 0.262 | 0.195 | 0.239 | 0.029 | 0.242 | 0.134 | 0.242 |

Robust standard errors in parentheses. * p < 0.1, ** p < 0.05, *** p < 0.01. Estimates of cut-off omitted due to space limitation.
